# Circ_0000284 Is Involved in Arsenite-Induced Hepatic Insulin Resistance Through Blocking the Plasma Membrane Translocation of GLUT4 in Hepatocytes via IGF2BP2/PPAR-γ

**DOI:** 10.3390/toxics12120883

**Published:** 2024-12-04

**Authors:** Shiqing Xu, Zhida Hu, Yujie Wang, Qiyao Zhang, Zhi Wang, Teng Ma, Suhua Wang, Xiaohui Wang, Li Wang

**Affiliations:** School of Public Health, Baotou Medical College, Inner Mongolia University of Science & Technology, Baotou 014040, China; 2022200097@stu.btmc.edu.cn (S.X.); 2021400116@stu.btmc.edu.cn (Z.H.); 2021400065@stu.btmc.edu.cn (Y.W.); 2020400068@stu.btmc.edu.cn (Q.Z.); 2022200104@stu.btmc.edu.cn (Z.W.); 102018924@btmc.edu.cn (T.M.); bt_wangsuhua@163.com (S.W.)

**Keywords:** arsenic exposure, diabetes mellitus, insulin resistance, circ_0000284, hepatotoxicity

## Abstract

Arsenic exposure can induce liver insulin resistance (IR) and diabetes (DM), but the underlying mechanisms are not yet clear. Circular RNAs (circRNAs) are involved in the regulation of the onset of diabetes, especially in the progression of IR. This study aimed to investigate the role of circRNAs in arsenic-induced hepatic IR and its underlying mechanism. Male C57BL/6J mice were given drinking water containing sodium arsenite (0, 0.5, 5, or 50 ppm) for 12 months. The results show that sodium arsenite increased circ_0000284 expression, decreased insulin-like growth factor 2 mRNA binding protein 2 (IGF2BP2) and peroxisome proliferator-activated receptor-γ (PPAR-γ), and inhibited cell membrane protein levels of insulin-responsive glucose transporter protein 4 (GLUT4) in the mouse livers, indicating that arsenic exposure causes liver damage and disruptions to glucose metabolism. Furthermore, sodium arsenite reduced glucose consumption and glycogen levels, increased the expression of circ_0000284, reduced the protein levels of IGF2BP2 and PPAR-γ, and inhibited GLUT4 protein levels in the cell membranes of insulin-treated HepG2 cells. However, a circ_0000284 inhibitor reversed arsenic exposure-induced reductions in IGF2BP2, PPAR-γ, and GLUT4 levels in the plasma membrane. These results indicate that circ_0000284 is involved in arsenite-induced hepatic insulin resistance through blocking the plasma membrane translocation of GLUT4 in hepatocytes via IGF2BP2/PPAR-γ. This study provides a scientific basis for finding early biomarkers for the control of arsenic exposure and type 2 diabetes mellitus (T2DM), and discovering new prevention and control measures.

## 1. Introduction

Diabetes mellitus (DM) can be influenced by genetic or environmental factors and is often characterised by hyperglycaemia and insulin abnormalities. The increasing prevalence of DM, particularly type 2 diabetes mellitus (T2DM), has put enormous pressure on healthcare resources, leading to a significant global public health problem [1,2]. Insulin resistance (IR) is the main aetiology and pathogenesis of T2DM. IR occurs due to the reduced sensitivity of the liver, fat, and other tissues to insulin. Notably, the liver is crucial for the maintenance of normal glucose homeostasis [3].

Arsenic is a toxic element found extensively in the environment, with an average concentration of about 5 mg/kg [4]. Although the World Health Organization (WHO) recommends a maximum arsenic concentration of 10 μg/L in drinking water, the recommendations for the maximum arsenic concentration in drinking water in over 40 countries exceed 10 μg/L [5]. About 200 million individuals globally are potentially exposed to elevated levels of arsenic in their drinking water [6]. Arsenic exposure is strongly associated with many human diseases, such as metabolic diseases, neurological diseases, skin diseases, and various cancers. In addition, arsenic exposure can lead to the occurrence of risk factors for cerebrovascular diseases, such as hypertension and T2DM [7].

Chronic arsenic exposure in humans can cause arsenic accumulation in the liver, affecting glucose metabolism, thus leading to IR and increasing the risk of T2DM [8]. A study showed that arsenic impairs glucose tolerance in mice after eight weeks of exposure to 50 mg/L inorganic arsenite in drinking water [9]. Another study showed that mice exposed to 20 ppm arsenite in drinking water for 12 months could develop IR [10]. Furthermore, the internal levels of arsenic in mice exposed to 50 ppm arsenite are comparable to the levels in highly exposed populations. Moreover, glucose tolerance is impaired in mice exposed to 50 ppm arsenite [9,11]. Therefore, a minimum of three doses should be selected to assess the dose–response correlation, adhering to established protocols for hazard identification and risk assessment [12]. In this study, 50 ppm, 5 ppm, and 0.5 ppm were selected as the maximum dose, medium dose, and low dose of sodium arsenite, respectively, to assess the effects of chronic sodium arsenite exposure on hepatic insulin resistance and its mechanism of action in mice.

Circular RNAs (circRNAs) are single-stranded non-coding RNAs with a covalent closed-loop structure. CircRNAs are involved in insulin resistance as microRNAs sponges or RNA-binding protein (RBP) sponges [13]. Circ_0000284 originates from the HIPK3 gene and has a length of 1099 nucleotides [14]. Circ_0000284 can cause hyperglycaemia and hepatic IR by sponging miR-192-5p [15]. Therefore, circ_0000284 may be involved in hepatic IR related to arsenic exposure.

The insulin-like growth factor 2 mRNA binding protein 2 (IGF2BP2) is an RBP involved in RNA localisation, stabilisation, and translation [16]. IGF2BP2 can participate in the development of liver cancer by binding to circRNAs [17]. Furthermore, IGF2BP2 was recognised as a T2DM-related gene as early as 2007, consistent with the findings of a meta-analysis of populations [18]. The liver-specific deletion of IGF2BP2 increases hepatic triglyceride accumulation and the risk of obesity, which are associated with IR-mediated T2DM [19,20]. However, it is unclear whether circ_0000284 promotes arsenic-induced hepatic IR by binding to IGF2BP2 and its downstream pathways.

Triglycerides accumulate in the liver when the transport rate of triglycerides from the liver to extrahepatic tissues is slowed down. This transport process may be related to the peroxisome proliferator-activated receptor-γ (PPAR-γ) [21]. In addition, hepatic PPAR-γ-deficient mice have a reduced glucose tolerance and insulin sensitivity [22]. The PPAR-γ activator ameliorates hepatic IR in arsenic-treated mice and HepG2 cells [23]. Glucose transporters (GLUTs) play a key role in insulin-stimulated glucose utilisation [24]. Although the insulin-responsive glucose transporter protein 2 (GLUT2) is the major GLUT isoform in the liver and is expressed at higher levels than insulin-responsive glucose transporter protein 4 (GLUT4), GLUT4 is a key regulator of systemic glucose homeostasis. GLUT4 is mainly expressed in adipose tissue, skeletal, and cardiac muscle cells [25]. However, it has been shown that GLUT4 is also involved in glucose metabolism in the liver, with IR significantly upregulating the expression of GLUT4 mRNA and protein in the liver, rather than GLUT2 expression [26]. The upregulating of GLUT4 can alleviate insulin resistance in the liver of mice [27]. In their study on the effects of metformin on hepatic glucose metabolism, Zhu et al. showed that the expression of GLUT4 in the liver is also significantly affected by metformin [28]. Furthermore, the downregulation of GLUT4 is the main pathological mechanism of hyperglycaemia induced by insulin resistance [29]. Therefore, we mainly investigated the expression of GLUT4 in hepatic IR induced by sodium arsenite. PPAR-γ and GLUT4 are elevated in diabetic rats. Notably, PPAR-γ regulates GLUT4 expression [30]. Although a specific GLUT4 knockdown leads to systemic glucose intolerance and IR in mouse muscle and liver tissues [31], the specific mechanism of action is unclear.

Herein, the role of circ_0000284 in arsenic exposure-induced hepatic IR is evaluated. The results reveal a novel molecular mechanism underlying hepatic IR and arsenic-induced T2DM. Therefore, this study provides a scientific basis for identifying the early biomarkers for controlling arsenic exposure and T2DM, and for discovering new preventive and curative measures for arsenic-induced T2DM.

## 2. Materials and Methods

### 2.1. Mouse Model for Arsenite Exposure

Eight-week-old male C57BL/6J mice were sourced from SPF Biotechnology Co., Ltd. (Beijing, China) (license No. 1100987757). All the animal experiments and housing conditions were approved by the Laboratory Animal Ethics Committee of Baotou Medical College (approval number No. 002, Baotou Medical College, 2021). The mice were stochastically divided into four groups (*n* = 12). Sodium arsenite (Sigma, Darmstadt, Germany) was diluted in drinking water to concentrations of 0, 0.5, 5, and 50 ppm, and the mice were allowed to drink the water freely for 12 months. Each mouse was fed with maintenance feed for rats and mice (SPEFO Biotechnology Co., Ltd., Beijing, China), which did not contain arsenic. The water consumption, food intake, and body weight of the mice were recorded weekly. After 12 months of sodium arsenite exposure, the mice were anaesthetised by an intraperitoneal injection of 1% pentobarbital sodium and then euthanised by cervical dislocation, and their livers were collected for further analysis.

### 2.2. Intraperitoneal Glucose Tolerance Tests (IPGTTs) and Insulin Tolerance Tests (ITTs)

The mice were fasted overnight after 12 months of arsenic exposure. The fasting blood glucose in the tail vein was measured using a blood glucose meter (Roche Diagnostics GmbH, Mannheim, Germany). The blood glucose levels were assessed at 15, 30, 60, and 120 min following the administration of a glucose solution (2 g/kg body weight dissolved in saline) via an intraperitoneal injection. The mice were given one day to recover after the IPGTTs. The mice were fasted for 4–6 h before the measurement of the ITTs. Insulin (0.5 IU/kg) was administered via an intraperitoneal injection, followed by blood glucose monitoring at 15, 30, and 60 min post-injection.

### 2.3. Liver Periodic Acid–Schiff (PAS) Staining

The fresh liver tissues were fixed in 4% paraformaldehyde, embedded in paraffin, and cut into 5 µm sections. The sections were dewaxed, rinsed with tap water for 2~3 min, then rinsed twice with distilled water, and placed in an oxidising agent at room temperature (25–30 °C) for 5~10 min. The samples were rinsed twice with distilled water, then put into a PAS staining solution (Solarbio Life Science, Beijing, China) in a dark place at room temperature (25–30 °C) for 10–20 min. The samples were put in a haematoxylin staining solution for 2 min to stain the nucleus. The sections were differentiated with acidic differentiation solution and dehydrated, made transparent, and sealed before visualisation using a microscope.

### 2.4. HE Staining

The fixed tissue samples were washed with running water, dehydrated with gradient alcohol, and embedded. The samples were cut into 3 μm thick paraffin slices and incubated at 65 °C for 4.5 h. The sections were deparaffinised and rehydrated through graded alcohols to water. The sections were then placed in a haematoxylin solution for 3 min, rinsed with running tap water for 5 min, and differentiated in 1% acid alcohol (1% HCl in 70% ethanol) for 10 s. After rinsing with running tap water for 1 min, the sections were placed in a blueing solution for 30 s to 1 min, and then rinsed again with running tap water for 5 min. Next, the sections were stained in an eosin dye solution for 5 min, dehydrated through graded alcohols, and cleared in xylene. Finally, the slides were sealed with a coverslip using a mounting medium, observed under a microscope, and photographed for analysis.

### 2.5. Cell Culture and Treatment

The human hepatocellular carcinoma cell line HepG2 was sourced from Pricella Life Science and Technology Co., Ltd. (Wuhan, China). The cell culture medium was DMEM (Gibco, Gaithersburg, MD, USA). The complete medium was supplemented with 10% foetal bovine serum (Gibco, Gaithersburg, MD, USA) and 1% antibiotics (100 IU/mL penicillin and 100 μg/mL streptomycin, Beyotime, Shanghai, China). The cells were cultured in a humidified incubator (Thermo Scientific, Waltham, MA, USA) at 37 °C with 5% carbon dioxide. The HepG2 cells were digested with trypsin when they grew to 80–90%, were re-laid evenly in well plates or cell culture dishes, and treated with different concentrations of the sodium arsenite culture solution for 24 h, followed by the addition of 100 nM insulin for 30 min. The cells were collected for subsequent experiments.

### 2.6. Cell Viability Assay

The cell viability was assessed using Cell Counting Kit 8 (CCK-8) reagent (Biosharp, Anhui, China). The cell concentration was determined using a cell counter. The cells (5000 per well) were inoculated into 96-well culture plates. The original medium was discarded after the cells attached to the wall. The cells were then treated with the medium containing different concentrations of sodium arsenite (0, 1, 2, 4, 6, 8, 10, 20, or 30 μM) for 24 h. The CCK-8 reagent (10 μL) was added to each well, and the absorbance was recorded at 450 nm. The cell viability was calculated as a percentage of the absorbance in the control wells.

### 2.7. Cell Transfection

The circ_0000284 inhibitors (si-circ_0000284) and si-circ_NC (si-circ_0000284 negative control) were sourced from IGEBio (Guangzhou, China). The cell transfection was completed using Liposome 2000 reagent (Invitrogen). The cells were inoculated into six-well plates, followed by the addition of the MEM solution containing si-circ_0000284 and the MEM solution with si-NC, mixed with the MEM solution containing the Liposome 2000 reagent, for transfection for 6 h. The cells were then incubated with the sodium arsenite solution for 24 h, and collected for further experiments.

### 2.8. Membrane Protein Extraction and GLUT4 Analysis

A Membrane Protein and Cytoplasmic Protein Extraction Kit (Beyotime, Shanghai, China) was used to extract the membrane proteins and plasma proteins. The HepG2 cells were collected via cell scraping, then centrifuged at 4 °C and 1200 rpm for 5 min. The supernatant was discarded, and the precipitate was retained. Moreover, the liver tissues were cut into pieces. Benzenesulfonyl fluoride (PMSF, Beyotime, Shanghai, China) was added to the cell and tissue samples, then homogenised with a homogeniser, centrifuged at 4 °C and 14,000× *g* for 30 min. The plasma protein was collected as the supernatant. Membrane Protein Extraction Reagent B was added to the precipitate and centrifuged at 14,000× *g* and 4 °C for 5 min to collect the membrane protein solution.

The total protein was obtained using a Total Protein extraction kit (Solarbio Life Science, Beijing, China). Protein lysate containing PMSF was added to the petri dish and scraped with a cell scraper after treating the cells according to each experimental protocol. Also, the liver tissue was ground, followed by the addition of protein lysate containing PMSF to prepare the tissue protein lysate. The protein lysates were lysed on ice for 30 min, then centrifuged at 4 °C and 12,000 rpm for 30 min. The supernatant was absorbed into a new centrifuge tube (total protein).

### 2.9. Western Blots

The protein concentration was measured using a BCA kit (APPLYGEN, Beijing, China) after protein extraction. Separation and concentration gels were prepared using an SDS-PAGE gel preparation kit (Solarbio Life Science, Beijing, China), up-sampled for electrophoresis, then transferred to a PVDF membrane. The membrane was blocked with 5% skimmed milk for 2 h and incubated with a primary antibody at 4 °C overnight. The membrane was also incubated with a secondary antibody for 1 h. Finally, the membrane was imaged after treatment with ECL reagent (APPLYGEN, Beijing, China). The primary antibodies for IGF2BP2, GLUT4, PPAR-γ, β-actin, Na/K-ATPase were obtained from Affinity, Abcam, Proteintech, SAB, and Bioss, respectively.

### 2.10. RNA Extraction and Quantitative Real-Time PCR (qRT-PCR) Analyses

The total RNA was extracted by adding 1 mL of TransZol Up reagent (TransGen Biotech, Beijing, China) to each petri dish. The total RNA extracts were reverse transcribed with GoScriptTMReverse Transcription System (Promega, Madison, WI, USA). The levels of circ_0000284 were detected using a PCR kit (Promega, Madison, WI, USA) on a LightCycler96 instrument (Roche, Basel, Switzerland). The data were normalised to β-actin via the 2^−ΔΔCt^ method [32]. The sequences of the specific primers are shown in Table 1.

### 2.11. Glycogen and Glucose Consumption Levels

For the glycogen, the cells were collected, followed by a cell count analysis (5–1 million cells). Glycogen extract (0.75 mL) was added to the collected cells and tissues, centrifuged, and the supernatant was discarded. The glycogen was assessed using a Glycogen Assay Kit (Solarbio, Beijing, China). For the glucose, the cells were collected, followed by a cell count analysis (5–1 million cells). The supernatant (1 mL) was added into boiling water for 10 min, followed by glucose analysis using a glucose assay kit (Solarbio, Beijing, China).

### 2.12. Statistical Analysis

SPSS 26.0 (IBM, Armonk, NY, USA) was used for the statistical analysis of the data, and GraphPad Prism 9.0 (GraphPad Software, San Diego, CA, USA) was used to plot the experimental results. One-way analyses of variance (one-way ANOVAs) were conducted to analyse the variations in the indicators across multiple groups, followed by pairwise comparisons between multiple groups using a Bonferroni correction. Each experiment was performed in at least three biological replicates and the results were expressed as the mean ± SD. Statistical significance was considered for a *p*-value < 0.05.

## 3. Results

### 3.1. Chronic Sodium Arsenite Exposure Causes Liver Injury and Hepatic IR in Mice

**Figure 1:**
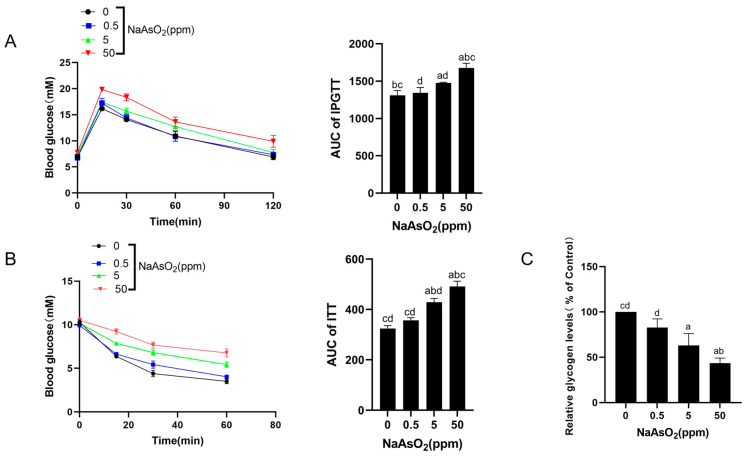

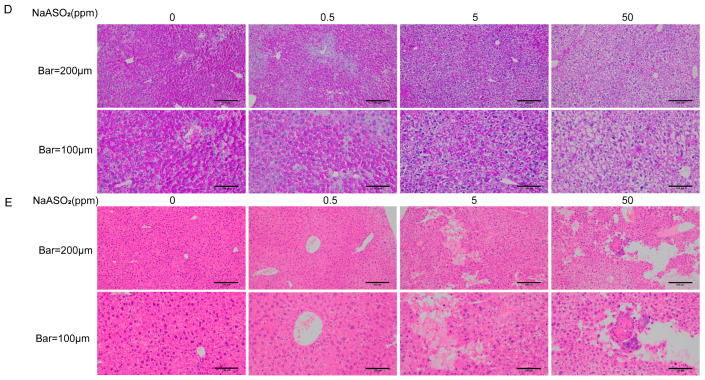
Chronic exposure to sodium arsenite causes liver injury and hepatic IR in mice. C57BL/6J mice were allowed to drink water containing 0, 0.5, 5, or 50 ppm sodium arsenite for 12 months. (**A**) IPGTTs showing the blood glucose concentrations at 0, 15, 30, 60, and 120 min in the mice. The area under the curve (AUC) was calculated based on the IPGTTs results. (**B**) ITT assays were performed to determine the blood glucose concentrations of the mice at 0, 15, 30, and 60 min. The AUC of the ITTs was calculated. (**C**) A glycogen assay kit was used to determine the glycogen concentration in each mouse’s liver. (**D**) The detection of hepatic glycogen changes by PAS staining. (**E**) The HE staining images of livers indicating the liver injury level. The data are presented as the mean ± SD, *n* = 3. ^a^: *p* < 0.05, compared with the 0 ppm NaAsO_2_ group; ^b^: *p* < 0.05, compared with the 0.5 ppm NaAsO_2_ group; ^c^: *p* < 0.05, compared with the 5 ppm NaAsO_2_ group; ^d^: *p* < 0.05, compared with the 50 ppm NaAsO_2_ group.

The mice were given drinking water containing 0, 0.5, 5, and 50 ppm sodium arsenite for 12 months and subjected to IPGTTs and ITTs. The results show that 5 and 50 ppm sodium arsenite reduced glucose tolerance in mice compared with the control mice, especially in the 50 ppm group (Figure 1A). Moreover, 5 and 50 ppm sodium arsenite significantly increased the glucose levels in the mice after an insulin injection compared with the control mice, thus reducing insulin sensitivity (Figure 1B). The glycogen levels decreased in the liver with increasing doses of sodium arsenite (Figure 1C,D). In addition, the HE staining showed significant liver damage in the high-dose group (Figure 1E). These results suggest that sodium arsenite causes liver injury and hepatic IR in mice.

### 3.2. Sodium Arsenite Increases Levels of Circ_0000284 Levels and Decreases IGF2BP2 Levels in Mouse Livers

**Figure 2:**
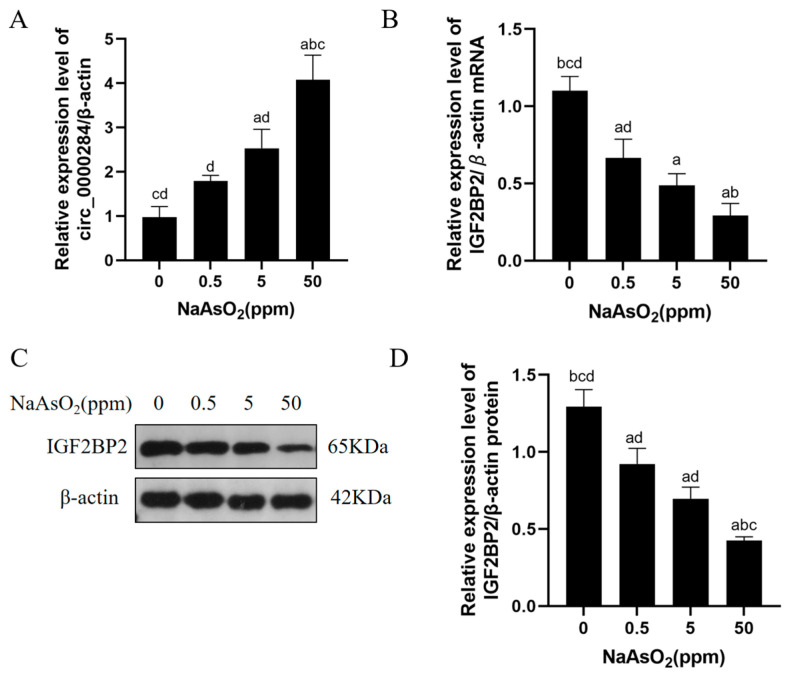
Chronic exposure to sodium arsenite induces increased levels of circ_0000284 and decreased levels of IGF2BP2 levels in livers of mice. C57BL/6J mice were allowed to drink water containing 0, 0.5, 5, or 50 ppm sodium arsenite for 12 months. Levels of circ_0000284 (**A**) and IGF2BP2 (**B**) in livers of mice were determined using qRT-PCR assay. (**C**) Western blots of protein bands and (**D**) relative protein levels of IGF2BP2 in livers of mice. Data are presented as mean ± SD, *n* = 3. ^a^: *p* < 0.05, compared with 0 ppm NaAsO_2_ group; ^b^: *p* < 0.05, compared with 0.5 ppm NaAsO_2_ group; ^c^: *p* < 0.05, compared with 5 ppm NaAsO_2_ group; ^d^: *p* < 0.05, compared with 50 ppm NaAsO_2_ group.

The levels of circ_0000284 and IGF2BP2, as well as the protein expression level of IGF2BP2, were evaluated to investigate whether circ_0000284 and IGF2BP2 play a role in arsenite-induced hepatic IR. Furthermore, the circ_0000284 levels were higher in the sodium arsenite-exposed mice than in the controls, especially in the 5 and 50 ppm groups (Figure 2A). Also, the mRNA and protein levels of IGF2BP2 were reduced in the arsenic-exposed mice in a dose–response manner compared with the controls (Figure 2B–D). These findings indicate that sodium arsenite increases circ_0000284 levels and reduces IGF2BP2 levels in mouse livers.

### 3.3. Arsenic Exposure Decreases PPAR-γ and Membrane GLUT4 Levels in Mice

**Figure 3:**
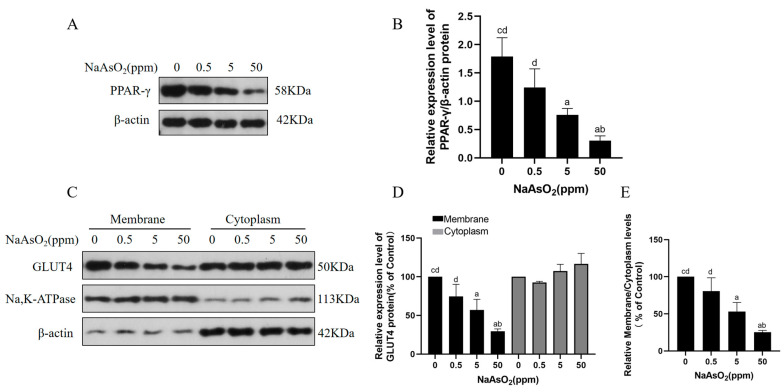
Chronic arsenic exposure decreases levels of PPAR-γ and levels of membrane GLUT4 in livers of mice. C57BL/6J mice were allowed to drink water containing 0, 0.5, 5, or 50 ppm sodium arsenite for 12 months. (**A**) Western blots of protein bands and (**B**) relative protein levels of PPAR-γ in livers of mice. (**C**) Western blots of protein bands and (**D**) relative protein levels of GLUT4 in cytoplasm and membrane of mice livers; β-actin served as internal reference for cytoplasm proteins, and Na and K-ATPase as internal references for membrane proteins. (**E**) Ratio of GLUT4 protein levels in membrane to cytoplasm. Data are presented as mean ± SD, *n* = 3. ^a^: *p* < 0.05, compared with 0 ppm NaAsO_2_ group; ^b^: *p* < 0.05, compared with 0.5 ppm NaAsO_2_ group; ^c^: *p* < 0.05, compared with 5 ppm NaAsO_2_ group; ^d^: *p* < 0.05, compared with 50 ppm NaAsO_2_ group.

The GLUT4 transporter protein is sequestered in specialised storage vesicles inside a cell under basal conditions. Increased circulating insulin activates an intracellular signalling cascade when glucose levels are elevated, ultimately leading to the translocation of GLUT4 from the storage region to the plasma membrane, thus promoting glucose uptake and maintaining glucose homeostasis [33]. Insulin resistance limits the transportation of GLUT4 transporter proteins to the cell membrane due to the lack of insulin stimulation [34]. The activation of PPAR-γ promotes GLUT4 translocation to the plasma membrane, whereas decreased PPAR-γ expression inhibits GLUT4 translocation to the plasma membrane, affecting cellular glucose homeostasis [35]. Herein, the levels of PPAR-γ and GLUT4 in the cytoplasm and membrane of the mouse livers after exposure to sodium arsenite (0, 0.5, 5, and 50 ppm) were assessed to investigate the roles of altered PPAR-γ expression and GLUT4 translocation in arsenite-induced hepatic insulin resistance. Sodium arsenite decreased the protein levels of PPAR-γ in the mouse livers (Figure 3A,B). Notably, sodium arsenite decreased the membrane protein levels of GLUT4 in the livers in a dose-dependent manner compared with the controls (Figure 3C,D). Moreover, sodium arsenite decreased the ratio of GLUT4 levels in the membrane proteins to cytoplasmic proteins compared with the controls, indicating the inhibition of GLUT4 translocation at the plasma membrane (Figure 3E). These findings suggest that sodium arsenite reduces the level of PPAR-γ expression and blocks the plasma membrane translocation of GLUT4 in hepatocytes.

### 3.4. Sodium Arsenite Decreases Levels of Glucose Consumption and Glycogen in Insulin-Treated HepG2 Cells

**Figure 4:**
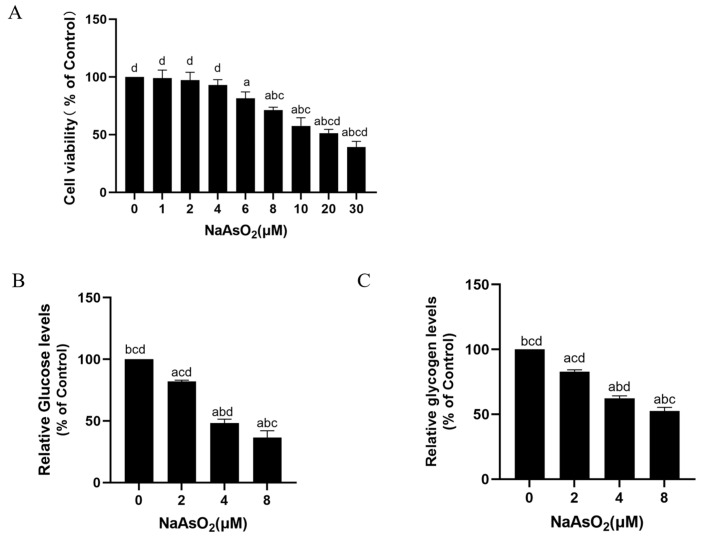
Sodium arsenite causes decreased levels of glucose consumption and glycogen in insulin-treated HepG2 cells. HepG2 cells were treated with 0, 1, 2, 4, 8, 10, 20, or 30 μM sodium arsenite for 24 h. (**A**) Cell viability was detected by CCK-8 assay. After HepG2 cells were treated with 0, 2, 4, or 8 μM sodium arsenite for 24 h, they were then treated for 30 min with 100 nM insulin. Glucose consumption (**B**) and glycogen levels (**C**) in HepG2 cells were measured by glucose assay kits and glycogen assay kits. Data are presented as mean ± SD, *n* = 3. ^a^: *p* < 0.05, compared with 0 μM NaAsO_2_ group; ^b^: *p* < 0.05, compared with 2 μM NaAsO_2_ group; ^c^: *p* < 0.05, compared with 4 μM NaAsO_2_ group; ^d^: *p* < 0.05, compared with 8 μM NaAsO_2_ group.

In this study, cellular experiments were used to further validate the effects of arsenic exposure on hepatic IR. First, CCK8 experiments were performed to select the appropriate poison dose for the HepG2 cells. Studies have shown that the viability of HepG2 cells does not significantly change at sodium arsenite concentrations ≤4 μM, and when the concentration of sodium arsenite is 8 μM, cell viability significantly decreases with statistical significance [23]. The results of this experiment are consistent with those of previous ones. Herein, the cells were treated with 0, 1, 2, 4, 6, 8, 10, 20, or 30 μM sodium arsenite, and the CCK-8 assay showed that the cell viability gradually decreased with an increasing sodium arsenite concentration (Figure 4A). Sodium arsenite < 2 μM did not significantly affect cell viability, while 4 μM sodium arsenite significantly decreased cell viability. The cell viability for 8 μM sodium arsenite decreased to about 70%. It has been shown that the ideal cell status and viability are >60–80% [36]. Therefore, the HepG2 cells were treated with 0, 2, 4, and 8 μM of sodium arsenite in the subsequent experiments. The HepG2 cells were treated with 0, 2, 4, or 8 μM sodium arsenite for 24 h, followed by a 100 nM insulin treatment for 30 min. The results show that the glucose consumption and glycogen levels of the HepG2 cells were lower in the arsenic-exposed cells than in the control cells in a dose-dependent manner (Figure 4B,C). These results suggest that sodium arsenite reduces insulin-dependent glucose consumption and glycogen levels in hepatocytes.

### 3.5. Sodium Arsenite Increases Levels of Circ_0000284 and Decreases Levels of IGF2BP2, PPAR-γ, and Membrane GLUT4 in Insulin-Treated HepG2 Cells

**Figure 5:**
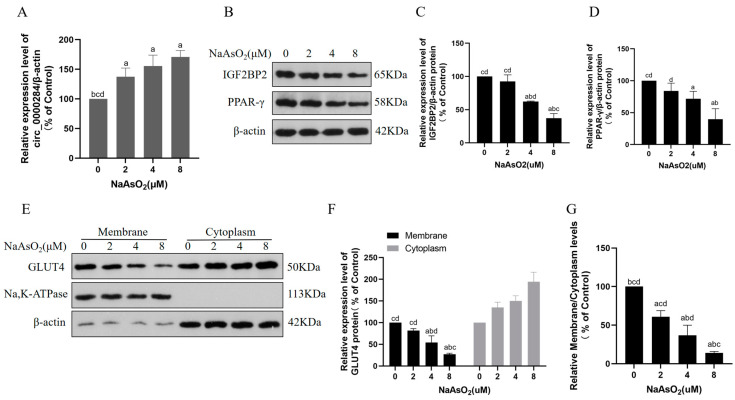
Sodium arsenite increases levels of circ_0000284 levels and decreases levels of IGF2BP2, PPAR-γ, and membrane GLUT4 in insulin-treated HepG2 cells. HepG2 cells were treated with 0, 2, 4, or 8 μM sodium arsenite for 24 h, and then treated for 30 min with 100 nM insulin. (**A**) Levels of circ_0000284 were quantified by qRT-PCR. (**B**) Western blots of protein bands and (**C**,**D**) corresponding relative protein levels of IGF2BP2 and PPAR-γ. (**E**) Western blots of protein bands and (**F**) relative protein levels of GLUT4 in cytoplasm and membrane; β-actin served as internal reference for cytoplasm proteins, and Na and K-ATPase as internal references for membrane proteins. (**G**) Ratio of GLUT4 expression levels in membrane proteins to cytoplasm proteins. Data are presented as mean ± SD, *n* = 3. ^a^: *p* < 0.05, compared with 0 μM NaAsO_2_ group; ^b^: *p* < 0.05, compared with 2 μM NaAsO_2_ group; ^c^: *p* < 0.05, compared with 4 μM NaAsO_2_ group; ^d^: *p* < 0.05, compared with 8 μM NaAsO_2_ group.

Similarly, the HepG2 cells were treated with 0, 2, 4, or 8 μM sodium arsenite for 24 h, followed by a 100 nM insulin treatment for 30 min. The levels of circ_0000284 were elevated in the sodium arsenite- and insulin-treated HepG2 cells (Figure 5A). In addition, the protein levels of IGF2BP2 and PPAR-γ were lower in the arsenic-exposed HepG2 cells than in the control cells in a concentration-dependent manner (Figure 5B–D). Compared with the control cells, the membrane protein levels of GLUT4 and the ratio of GLUT4 levels in the membrane proteins to cytoplasmic proteins were reduced, indicating the inhibition of GLUT4 translocation at the plasma membrane (Figure 5E–G). These results suggest that arsenic exposure increases circ_0000284 levels, decreases IGF2BP2 and PPAR-γ expression levels, and blocks the plasma membrane translocation of GLUT4 in hepatocytes.

### 3.6. Inhibition of Circ_0000284 Blocks Sodium Arsenite-Induced Increases in Circ_0000284 Levels and Decreases in Glucose Consumption and Glycogen Levels in Insulin-Treated HepG2 Cells

**Figure 6:**
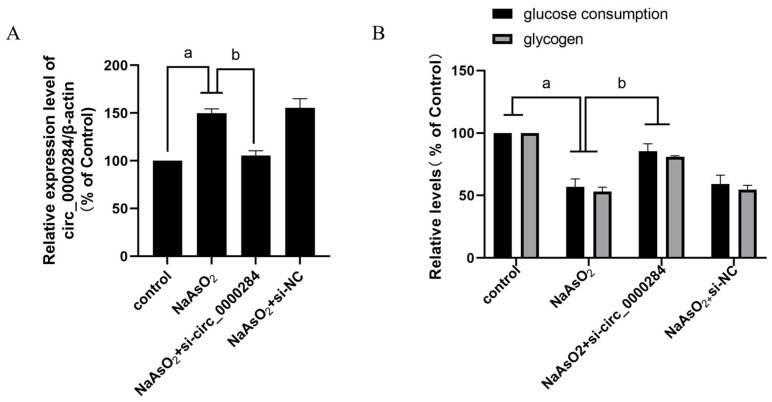
Inhibition of circ_0000284 blocks sodium arsenite-induced increases in circ_0000284 levels and decreases in glucose consumption and glycogen levels in insulin-treated HepG2 cells. HepG2 cells were transfected with 0 or 50 nM si-circ_0000284 or si-circ_NC for 6 h, followed by treatment with 0 or 8 μM sodium arsenite for 24 h, respectively, and then treated for 30 min with 100 nM insulin. (**A**) Levels of circ_0000284 in HepG2 cells were detected by qRT-PCR assay. (**B**) Glucose consumption and glycogen levels in HepG2 cells were measured by glucose assay kits and glycogen assay kits. Data are presented as mean ± SD, *n* = 3. ^a^: *p* <0.05, compared with HepG2 cells without arsenite treatment; ^b^: *p* <0.05, compared with si-circ_0000284-treated HepG2 cells.

The glucose consumption and glycogen levels were examined after the HepG2 cells were transfected with 0 or 50 nM si-circ_0000284 or si-circ_NC for 6 h to determine the role of circ_0000284 in hepatic IR in HepG2 cells exposed to arsenite. Notably, the cells were treated with 0 or 8 μM sodium arsenite for 24 h, followed by a 100 nM insulin treatment for 30 min. The downregulation of circ_0000284 levels by si-circ_0000284 reversed the sodium arsenite-induced effect on the circ_0000284 levels, glucose consumption, and glycogen levels in the insulin-stimulated HepG2 cells (Figure 6), indicating that circ_0000284 is involved in arsenite-induced IR in hepatocytes.

### 3.7. Inhibition of Circ_0000284 Blocks Sodium Arsenite-Induced Decreases in IGF2BP2, PPAR-γ, and Membrane GLUT4 Levels in Insulin-Treated HepG2 Cells

**Figure 7:**
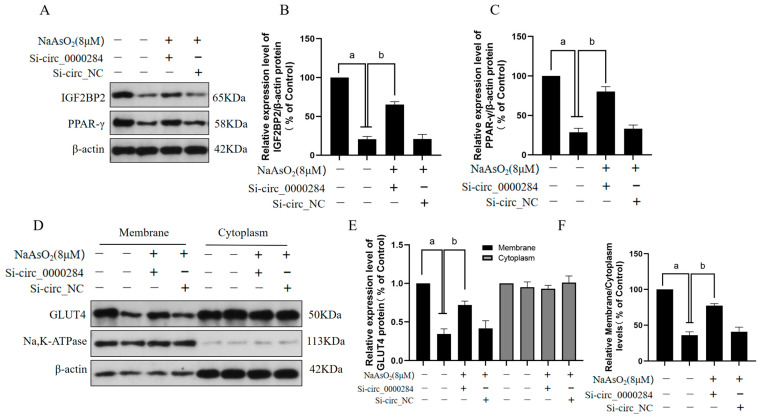
Inhibition of circ_0000284 blocks sodium arsenite-induced decreases in IGF2BP2, PPAR-γ, and membrane GLUT4 levels in insulin-treated HepG2 cells. After HepG2 cells were transfected with 0 or 50 nM si-circ_0000284 or si-circ_NC for 6 h, and then with 0 or 8 μM sodium arsenite for 24 h, respectively, they were then treated for 30 min with 100 nM insulin. (**A**) Western blots of protein bands and (**B**,**C**) corresponding relative protein levels of IGF2BP2 and PPAR-γ. (**D**) Western blots of protein bands and (**E**) corresponding relative protein levels of GLUT4 in cytoplasm and membrane; β-actin served as internal reference for cytoplasm proteins, and Na and K-ATPase served as internal references for membrane proteins. (**F**) Ratio of GLUT4 expression levels in membrane proteins to cytoplasm proteins. Data are presented as mean ± SD, *n* = 3. ^a^: *p* <0.05, compared with HepG2 cells without arsenite treatment; ^b^: *p* <0.05, compared with si-circ_0000284-treated HepG2 cells.

The effects of circ_0000284 on sodium arsenite-induced decreases in the levels of IGF2BP2, PPAR-γ, and membrane GLUT4 in insulin-treated HepG2 cells were examined to further investigate the mechanisms of circ_0000284 in hepatic IR in HepG2 cells exposed to sodium arsenite. HepG2 cells were cultured in a medium containing arsenite (0 or 8 μM) for 24 h after transfection with si-circ_0000284 or si-circ_NC, followed by an insulin (100 nM) treatment for 30 min. The results show that circ_0000284 downregulation after treatment with si-circ_0000284 alleviated the arsenite-induced decrease in the protein levels of PPAR-γ and IGF2BP2 (Figure 7A–C). Furthermore, the circ_0000284 inhibition blocked the decrease in GLUT4 levels at the membrane in the arsenic-induced HepG2 cells (Figure 7D–F). These findings suggest that circ_0000284 inhibitors can reverse the reduction in IGF2BP2 and PPAR-γ expression levels and the blockade of the plasma membrane translocation of GLUT4 in hepatocytes exposed to arsenic.

## 4. Discussion

Currently, several high-arsenic areas exist in all countries around the world, and arsenic exposure from drinking water can cause severe health problems in humans. Long-term arsenic exposure can induce liver fibrosis, nonalcoholic fatty liver disease, diabetes, and other diseases [37,38,39]. Arsenic exposure has been linked to T2DM, and T2DM is correlated with IR and insufficient insulin secretion [40,41,42]. The liver is the main target organ of arsenic metabolism. In recent years, an increasing number of studies have demonstrated that arsenic exposure may trigger hepatic IR, reduce the sensitivity of liver cells to insulin, hinder the uptake of glucose by liver cells, affect liver glycogen synthesis, and elevate blood sugar levels, which increase the risk of diabetes [43]. We found that arsenic exposure decreased the glycogen content and reduced the glucose consumption in HepG2 cells, inducing liver damage in mice, as well as impaired glucose tolerance and insulin sensitivity, leading to hepatic insulin resistance. However, the molecular mechanism leading to arsenic-induced hepatic IR is not well understood. In this study, arsenic exposure models of mice and HepG2 cells were established to explore the epigenetic mechanism of arsenic-induced hepatic IR.

In recent years, the role of ncRNA in the progression of diabetes following arsenic exposure has been extensively investigated in vivo [44]. Arsenic exposure can alter the expression of various proteins by affecting changes in ncRNA, causing IR [10]. However, the role of circRNAs in IR induced by arsenic exposure is not fully understood. Circ_0000284 can regulate apoptosis, proliferation, migration, and angiogenesis, as well as influence the development of cardiovascular diseases [45]. Circ_0000284 may also promote the proliferation of hepatobiliary carcinoma and breast cancer cells [14,46]. In this study, we showed that circ_0000284 expression was elevated in hepatic IR induced by arsenic exposure. This is consistent with the results obtained from the clinical experiments of Su et al. that, through a circRNA chip analysis of the peripheral blood of T2DM patients and a control group, revealed that the expression level of circ_0000284 in the T2DM group was significantly higher than that in the control group [47]. These results indicate that circ_0000284 is involved in the occurrence of hepatic IR induced by arsenic exposure.

CircRNAs have been implicated in the regulation of β cell function, IR, adipocyte function, inflammation, and oxidative stress by interacting with RBP [13]. IGF2BP2, as an RBP regulating multiple biological processes, is a T2DM-related molecule that regulates cellular metabolism in a variety of cell types. The expression of IGF2BP2 has been linked to liver fibrosis, T2DM, and cancer [21,48]. Furthermore, Greenwald found that the reduced activity of the IGF2BP2 homolog, Imp2, in mouse islets, impaired islet chromatin accessibility and glucose-stimulated insulin secretion [49]. Here, we show that IGF2BP2 serves as the downstream target of circ_0000284 through gene prediction tools. The results of this study show that with increasing levels of arsenic exposure, the expression level of circ_0000284 in liver cells increases, while the expression level of IGF2BP2 decreases, leading to liver IR. Furthermore, the silencing of circ_0000284 in hepatocytes results in the upregulation of the expression of IGF2BP2.

PPAR-γ is a ligand-activated nuclear transcription factor associated with adipose differentiation, obesity, and insulin resistance. PPAR-γ can affect glucose uptake in IR-mediated tissues and maintain glucose homeostasis [50]. Increased PPAR-γ expression is associated with improved insulin sensitivity and reduced insulin resistance [51]. In bone marrow-derived macrophages (BMDMs), IGF2BP2 can bind to fragments of PPAR-γ mRNA, thereby enhancing its stability [52]. In the liver tissues of WT mice, PPAR-γ mRNA is enriched in IGF2BP2 immunosuppression samples, and IGF2BP2 mediates the post-transcriptional regulation of PPAR-γ in the liver [53]. The deletion of IGF2BP2 is linked to a marked reduction in PPAR-γ mRNA levels [19]. In our study, as the level of arsenic exposure increased, the expression level of IGF2BP2 in the liver cells decreased, and the expression level of PPAR-γ also decreased. Consequently, we have established a correlation between IGF2BP2 and PPAR-γ. And, after knocking down circ_0000284 in the hepatocytes, the expression level of IGF2BP2 increased, and the level of PPAR-γ also increased. These findings indicate that circ_0000284 regulates IGF2BP2/PPAR-γ expression, influencing liver insulin resistance following exposure to arsenic.

GLUT4 serves as a pivotal regulator of the overall glucose balance within the body. Insulin reduces blood glucose levels by inhibiting hepatic glucose production and stimulating glucose uptake in tissues through GLUT4 [54]. Insulin binds to the receptors on the cell membrane, triggering a cascade of intracellular signalling events that result in the translocation of GLUT4 to the cell surface. This mechanism facilitates the absorption and transport of glucose into cells, maintaining glucose homeostasis. The aberrant expression of GLUT4 affects glucose homeostasis [55]. Research has demonstrated that the activation of PPAR-γ by insulin modulates GLUT4 translocation and lowers cellular glucose levels [56]. Furthermore, a diet high in sugar can trigger insulin resistance by decreasing the protein expression of PPAR-γ and GLUT4 within liver tissue, thereby disrupting glucose homeostasis [28]. While PPAR-γ’s role in arsenic-induced hepatic IR and its regulation of GLUT4 expression requires further investigation, our study confirms a dose-dependent decrease in PPAR-γ expression in hepatocytes exposed to arsenic. Simultaneously, the presence of GLUT4 in the cell membrane is decreased, suggesting GLUT4 translocation. The downregulation of circ_0000284 counteracts this effect. These findings suggest that circ_0000284 inhibits the translocation of GLUT4 to the plasma membrane of hepatocytes by modulating the IGF2BP2/PPAR-γ pathway, facilitating hepatic insulin resistance induced by arsenic exposure.

This study still has the following limitations. Firstly, we only studied the role of circ_0000284 in arsenic-induced insulin resistance in HepG2 cells, and there was a lack of an inhibitor group in vivo, which is a flaw of our study. In our next study, we will construct an animal model with the knockdown of circ_0000284 to further confirm its role in arsenite-induced hepatic insulin resistance. Secondly, we did not initially test for arsenic levels in the livers, which represents a lapse in our experimental design. Moving forward, we will measure the arsenic content in the liver tissues of mice.

## 5. Conclusions

In summary, arsenic exposure increases circ_0000284 levels in mouse livers and insulin-treated hepatocytes. Arsenic exposure affects insulin-dependent glucose consumption and glycogen accumulation by altering the expression level of circ_0000284 in hepatocytes, and affecting the expression of IGF2BP2, PPAR-γ, and GLUT4 in membrane proteins. These results indicate that circ_0000284 is involved in arsenite-induced hepatic IR through blocking the plasma membrane translocation of GLUT4 in hepatocytes via IGF2BP2/PPAR-γ. This study provides a scientific basis for finding early biomarkers for the control of arsenic exposure, offering insights into identifying the biomarkers for T2DM and for the prevention and treatment of arsenic poisoning.

## Figures and Tables

**Table 1:** Sequences of qRT-PCR primers and siRNAs.

Genes	F (5′→3′)	R (5′→3′)
hsa-circ_0000284	CGGCAGCCTTACAGGGTTAA	GACCAAGACTTGTGAGGCCA
mmu-circ_0000284	TGTTGGIGGATCCTGTTCGC	GACCAAGACTTGTGAGGCCA
IGF2BP2	GTCCTACTCAAGTCCGGCTAC	CATATTCAGCCAACAGCCCAT
hsa-β-actin	CAGATGIGGATCAGCAAGCAGGAG	GTCAAGAAAGGGTGTAACGCAACTAAG
mmu-β-actin	GGCTGTATTCCCCTCCATCG	CCAGTTGGTAACAATGCCATGT
Si-circ_0000284	GUACUACAGGUAUGGCCUGdTdT	GAGGCCAUACCLGUAGUACdTdT
Si-circ_NC	UUCUCCGAACGUGUCACGUTT	ACGUGACACGUUCGGAGAATT

## Data Availability

The data that support the findings of this study are available from the corresponding author upon reasonable request.
